# Socioeconomic disparities in depression risk: Limitations of the moderate effect of physical activity changes in Korea

**DOI:** 10.1371/journal.pone.0314930

**Published:** 2025-02-04

**Authors:** Su Kyoung Lee, Yong Jin Kwon

**Affiliations:** 1 Institute of Health and Environment Graduate School of Public Health Seoul National University, Seoul, Republic of Korea; 2 Department of Public Healthcare Center, Seoul National University Hospital, Seoul, Republic of Korea; Universiti Malaya, MALAYSIA

## Abstract

This study investigates the influence of changes in physical activity (PA) patterns on depression risk across different socioeconomic statuses (SES) in Korea. Utilizing National Health Insurance Data (NHID) from over 1.2 million individuals during 2013–2016, we matched medical aid beneficiaries with health insurance beneficiaries, excluding those with prior depression or incomplete PA data. Changes in moderate-to-vigorous PA (MVPA) were categorized into 16 groups, and depression incidence was tracked from 2019 to 2021. After adjustment, medical aid beneficiaries consistently showed higher risks of depression compared to health insurance enrollees with the same physical activity (PA) change patterns. For those consistently inactive, the risk was 1.68 times higher (aOR, 1.68; 95% CI, 1.37–2.05). Those who increased PA from inactivity to moderate-to-vigorous activity 3–4 times per week had a 3.33 times higher risk (aOR, 3.33; 95% CI, 1.72–6.43). Additionally, the risk was 2.64 times higher for those increasing from 1–2 times to ≥5 times per week (aOR, 2.64; 95% CI, 1.35–5.15), and 2.83 times higher for those consistently engaging in PA 3–4 times per week (aOR, 2.83; 95% CI, 1.35–5.94). Across the overall PA patterns, medical aid beneficiaries consistently faced higher depression risks, with risk increases of 1.80 times for increased activity, 1.68 times for continuous inactivity, and 1.34 times for decreased activity compared to health insurance beneficiaries with the same PA change patterns. However, in the consistently very active group, no significant difference in the risk of depression was observed between the two groups. Limitations include potential bias in self-reported PA and the NHIS data not fully capturing depression severity. The findings underscore the significant impact of SES on mental health, with consistently high PA levels potentially mitigating SES-related depression risk.

## Introduction

In 2019, mental health disorders comprised 16% of global Disability-Adjusted Life Years (DALYs), totaling 418 million DALYs [1]. Before 2020, depression, which affected more than 280 million people globally, was one of the major challenges in the top 25 global health burdens [2,3]. Depression poses a significant threat to both disability and mortality, elevating the risks of suicide and other physical ailments [2]. Therefore, it is crucial to understand and address the factors that contribute to the development and maintenance of depression. One of them could be physical activity (PA), which has been shown to have beneficial effects on mental and physical health [4]. However, PA levels are often low among people with low socioeconomic status (SES), low income, and social disadvantage, who are also more likely to experience depression [5]. Emerging evidence suggests that depression disproportionately affects people with low SES who face multiple barriers to accessing adequate mental health care and engaging in health-promoting behaviors [6,7].

Numerous studies from observational and experimental settings have demonstrated that PA can have positive effects on mental health outcomes, such as reducing the risk and severity of depression, at the population level, across different age groups [4,8,9]. However, there is a complex association between PA and depression, which may be influenced by various factors, such as socioeconomic status (SES), sedentary time, and well-being [10,11]. A recent umbrella review revealed that Compared to those who are less disadvantaged, those who are socioeconomically deprived have lower levels of PA and higher rates of health issues [12]. Moreover, some studies have suggested that PA may have a differential impact on depression depending on the SES level, with either stronger, weaker, or no significant interaction effects [13]. A cross-sectional study of older adults in Germany found that self-reported PA was negatively associated with depression among both men and women, and that this association was stronger for low-SES groups than high-SES groups [14]. Meanwhile, a study involving 24,102 adults in Korea revealed that the combination of low SES and metabolic syndrome was associated with a higher prevalence of depression, and PA did not moderate this association [15]. Despite these research findings, studies on whether physical activity can mediate the gap in depression risk across different SES groups are still limited.

To address this evidence gap, we examined the association between changes in PA patterns before a depression diagnosis and the risk of depression across different SES groups. Using a population-based approach, this study compares the risk of depression between socioeconomically vulnerable Medicare beneficiaries and those with health insurance from the customized National Health Information Database (NHID), and explores the less-researched area of whether varying levels of physical activity can mitigate the depression risk between these groups.

## Materials and methods

### Study population

This study utilized data obtained from a customized National Health Information Database (NHID) provided by the Korean National Health Insurance Service (NHIS). The NHIS provides health insurance services to approximately 97% of South Korea’s population and includes data on all Medical Aid beneficiaries except foreigners in the NHID. This research was supported by the NHID’s customized claims data, encompassing comprehensive census information, thus enhancing the generalizability and reliability of the study findings. Notably, South Korea maintains a distinct health coverage system for individuals with low income, where their medical information is aggregated by the NHIS. Medical Aid beneficiaries, qualifying under the Medical Care Assistance Act [16] in 2017 and 2018, were identified for this study, while those covered by National Health Insurance belong to separate and distinct groups without any overlap. The NHIS collected health insurance claims inclusive of medical screenings, treatments, and prescribed medications.

**Fig 1:**
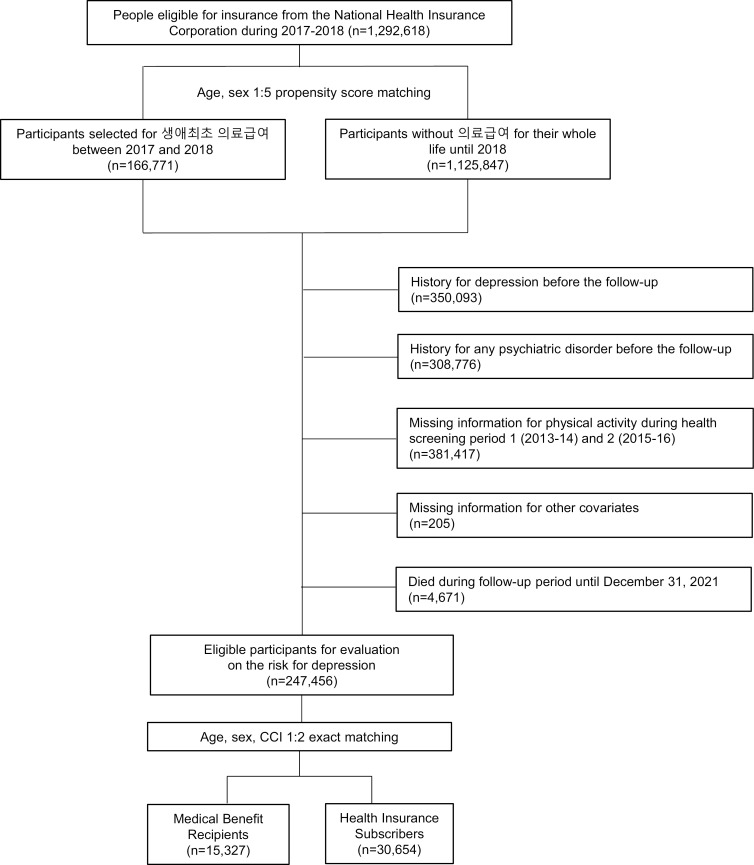
Flow diagram for the inclusion of the study population.

This study initially included 1,292,618 participants. The case group included individuals who transitioned to medical aid beneficiaries between 2017 and 2018, while the control group, matched at a 1:5 ratio based on age, sex, and regional propensity scores, included those who did not receive medical benefits until 2018. The case population comprised 166,771 medical aid beneficiaries, while 1,125,847 participants were in the control group. To create a cohort of individuals with complete information, those with a history of depression (n = 350,093), other mental health conditions (n = 308,776), missing PA survey data (n = 381,417), or missing covariate data (n = 205) were excluded. Additionally, those who died during follow-up were also excluded (n = 4,671) to avoid confounding by mortality. These exclusions resulted in 247,456 eligible participants. To minimize bias, exact matching in a 1:2 ratio was implemented based on age, sex, and Charlson Comorbidity Index (CCI). A total of 15,327 medical aid beneficiaries and 30,654 health insurance beneficiaries were followed from January 1, 2019, to December 31, 2021, to track depression events (Fig 1). This study was approved by the Institutional Review Board of Seoul National University Hospital Biomedical Research Institute (No.: E-2301-049-1394), and informed consent was waived due to the anonymized and confidential nature of the database provided for research purposes.

### Classification of change in physical activity

During each national health screening, individuals in the NHIS cohort completed self-administered questionnaires regarding their PA and other lifestyle habits. Utilizing the NHIS survey data, we analysed the frequency of moderate (≥30 minutes per day; like brisk walking, dancing, gardening) or vigorous (≥20 minutes per day; such as running, fast cycling, aerobic exercises) activities per week during the biennial health screenings in 2013–14 and 2015–16. This analysis aimed to track changes in MVPA (moderate-to-vigorous physical activity) among the participants.

For our main analysis, we established different categories to indicate the increase in MVPA from the period Ⅰ (2013–2014), where participants were physically inactive, to the period Ⅱ (2015–2016). These categories include: (i) continuously physically inactive, (ii) transitioning from being inactive to engaging in 1–2 times of MVPA weekly, (iii) transitioning from inactive to 3–4 times of MVPA weekly, and (iv) transitioning from inactive to more than five times of MVPA weekly. Likewise, we formulated classifications to represent the reduction in MVPA during the intervals between two consecutive biennial health screening periods as outlined below: (i) consistently engaging in ≥5 of MVPA per week, (ii) decreasing from ≥5 of MVPA per week to 3–4 times of MVPA per week, (iii) decreasing from ≥5 of MVPA per week to 1–2 times of MVPA per week, and (iv) decreasing from ≥5 of MVPA per week to being physically inactive. The accuracy and consistency of the PA questionnaire used in the NHIS cohort were detailed in a previous study [17]. Furthermore, we converted MVPA to metabolic equivalent of task (MET) based on energy costs for each PA. Total MET-min/week was calculated by multiplying MET values with frequency and duration. We assigned a MET value of 4.0 to MPA and 8.0 to VPA, as determined by a previous study [18]. MVPA levels were categorized as: (1) physically inactive (0 MET min/week), (2) insufficiently active (1 to < 500 MET min/week), (3) active (500 to < 1000 MET min/week), and (4) highly active (≥1000 MET min/week).

### Follow-up for depression outcomes

The primary outcome of this research was to determine the occurrence of new cases of depression within the monitoring timeframe spanning from January 1, 2019, to December 31, 2021. The definition of incident depression relied on specific codes outlined in the International Classification of Diseases, Tenth Revision (ICD-10), specifically F32-F33, in addition to reviewing prescription records for antidepressant medications. The classification using these ICD-10 codes for depression was derived from a study that elucidated the operational definition of depression in the Korea NHIS database [19].

### Key variables for adjustment and subgroup analyses

Pertinent data within the NHIS cohort database were gathered to identify primary variables utilized for adjustment and subgroup analyses. Socioeconomic factors such as age, sex, insurance premium (indicative of income status), place of residence (used as a measure for urbanization level), and the presence of disability, along with medical conditions encompassing medication use, as well as CCI calculated using cumulative medical claims records until the follow-up period), were sourced respectively from the NHIS insurance eligibility database and medical claims database [20]. Additional lifestyle behaviors like cigarette smoking and alcohol consumption, and clinical laboratory findings (comprising body mass index, blood pressure, total cholesterol, and fasting serum glucose) were identified using data sourced from the NHIS national health screening database. Past medical history such as diabetes (identified by a fasting serum glucose level of ≥126 mg/dL or the usage of antidiabetic medication), hypertension (defined by a systolic blood pressure of ≥140 mmHg or diastolic blood pressure of ≥90 mmHg, or the use of antihypertensive medications), and hypercholesterolemia (noted by a total cholesterol level of ≥250 mmHg or the usage of statin medication) were determined through biochemical tests and medication usage, aligning with methodologies described in earlier studies [21].

### Statistical analyses

The study’s follow-up period started on January 1, 2019, and concluded on December 31, 2021. Throughout this follow-up, each participant in the analytical cohort was monitored until the occurrence of their initial depression event. Those who enrolled in the study were continually observed and followed until December 31, 2021 or death, whichever happened earliest. The characteristics of participants at the outset of the study were calculated using n (%) for categorical variables and means [standard deviations (SDs)] for continuous variables. We calculated the event/total denoting the number of new cardiovascular events per 1000 person-years (PY), considering both the total cardiovascular events and PY within each group based on changes in MVPA. The age-adjusted and multivariate-adjusted logistic regression analysis was utilized to compute the adjusted odds ratio (aOR) along with the corresponding 95% confidence interval (CI). In the multivariate regression, odds were computed with adjustments for various key factors such as age, gender, income rank, BMI, smoking status, alcohol consumption, history of hypertension, diabetes, dyslipidemia, and CCI. Utilizing the Cox proportional hazards model, we computed the hazard ratio (HR) and 95% confidence intervals (CIs) to examine depression outcomes to changes in MVPA between two consecutive biennial health screening periods (2013–2014 to 2015–2016). Initially, we formulated a minimally adjusted model by incorporating age and sex as the variables for adjustment. Adjusted hazard ratios (HRs) and their respective 95% confidence intervals (CIs) concerning depression outcomes were derived by adjusting for the following factors within the Cox regression model: age, sex, place of residence, insurance premium, body mass index, systolic blood pressure, total cholesterol, fasting serum glucose, cigarette smoking, alcohol consumption, medication usage, and CCI. To evaluate the appropriateness of the Cox proportional hazards models, we conducted graphical assessments to verify the proportionality assumption.

In the primary analysis, we investigated changes in MVPA by concentrating on physically inactive individuals and those engaging in MVPA for ≥5 times per week during the period Ⅰ (2013–2014). Within this analysis, we used participants categorized as consistently physically inactive and those consistently engaging in MVPA for ≥5 times per week as references. Previous studies regarding financial issues with depression events suggested that change in depression state is due to an earlier change in socioeconomic circumstance, and current changes have no effect [22,23]. Therefore, we preceded the medical aid events and followed up for any depression incidence. For subgroup analyses, we categorized participants based on various factors: age (<65, ≥65), sex (male and female), presence of disability (with and without), cigarette smoking history (never, past smoker, and current smoker), weight status (obese and non-obese), diabetes, hypertension, hypercholesterolemia (yes and no), and comorbidity (CCI < 2 and ≥2). In secondary analyses, within subgroups of participants consistently engaging in 1–2 and 3–4 times of MVPA per week, we examined how changes in MVPA relate to the incidence of depression events. All data collection, processing, and statistical analyses in this study were conducted using SAS 9.4 (SAS Institute, Cary, NC, USA). Statistical significance for all analyses was set at a two-sided *P* value < 0.05.

## Results

### Baseline characteristics

The analysis cohort consisted of a total of 45,981 individuals, comprising 15,327 medical aid beneficiaries and 30,654 health insurance beneficiaries through 1:2 matching. the mean (SD) age was 59.6 (13.1), with 17,564 medical aid beneficiaries and 8,782 health insurance beneficiaries being male (57.3%). Roughly half of the individuals during both biennial health screening from the period Ⅰ (2013–2014) to period Ⅱ (2015–2016) reported being physically inactive. This trend was observed among both medical aid beneficiaries (47.5% in the period Ⅰ and 53.2% in the period Ⅱ) and health insurance beneficiaries (43.1% in the period Ⅰ and 50.2% in the period Ⅱ). The PA patterns between period Ⅰ and period Ⅱ revealed the following findings: (1) Continuously physically inactive individuals numbered 4,585 (32.8%) among medical aid beneficiaries and 7,812 (28.2%) among health insurance beneficiaries; (2) Those who increased MVPA amounted to 4,210 individuals (30.1%) among medical aid beneficiaries and 8,766 individuals (31.7%) among health insurance beneficiaries; (3) Decreased MVPA included 3,817 individuals (27.3%) among medical aid beneficiaries and 7,866 individuals (28.4%) among health insurance beneficiaries; (4) Individuals consistently physically active accounted for 1,368 (9.8%) among medical aid beneficiaries and 3,224 (11.7%) among health insurance beneficiaries. Other characteristics between medical aid beneficiaries and health insurance beneficiaries are detailed in Table 1.

**Table 1:** Descriptive characteristics of the study participants.

Variables	Medical aid beneficiaries^1^ (n = 15327)	Health insurance beneficiaries^2^ (n = 30654)
**Age, years**	56.9 (13.1)	56.9 (13.1)
**Sex, n (%)**		
Male	17564 (57.3)	8782 (57.3)
Female	13090 (42.7)	6545 (42.7)
**Physical activity at health screening period I (2013–2014)**		
**MVPA per week**		
Physically inactive	7278 (47.5)	13202 (43.1)
1–2 times	2737 (17.9)	5841 (19.1)
3–4 times	2200 (14.4)	4700 (15.3)
≥5 times	3112 (20.3)	6911 (22.6)
**MET per week**		
Physically inactive	7278 (47.5)	13202 (43.1)
0–500	5148 (33.6)	10973 (35.8)
500–1000	2107 (13.8)	4856 (15.8)
≥1000	794 (5.2)	1623 (5.3)
**Physical activity at health screening period II (2015**–**2016)**		
**MVPA per week**		
Physically inactive	5288 (53.2)	14977 (50.2)
1–2 times	3266 (32.9)	10428 (35.0)
3–4times	994 (10.0)	3201 (10.7)
≥5 times	389 (3.9)	1205 (4.0)
**MET per week**		
Physically inactive	7030 (45.9)	12745 (41.6)
0–500	5122 (33.4)	11016 (35.9)
500–1000	2409 (15.7)	5132 (16.7)
≥1000	766 (5.0)	1761 (5.7)
**Household income, n (%)**		
1^st^ quartile	6015 (39.2)	5933 (19.4)
2^nd^ quartile	2709 (17.7)	5490 (17.9)
3^rd^ quartile	2937 (19.2)	8115 (26.5)
4rd quartile (highest)	3666 (23.9)	11116 (36.3)
**Baseline comorbidities, n (%)**		
Hypertension	7135 (46.6)	13092 (42.7)
Diabetes	2786 (18.2)	4748 (15.5)
Dyslipidemia	5253 (34.3)	9901 (32.3)
**Charlson comorbidity index, n (%)**		
0	8404 (54.8)	16808 (54.8)
1	4214 (27.5)	8428 (27.5)
≥2	2709 (17.7)	5418 (17.7)
**Body mass index, n (%)**		
<18.5 kg/m2	464 (3.0)	788 (2.6)
18.5–23.0 kg/m2	5165 (33.7)	10296 (33.6)
23.0–25.0 kg/m2	3812 (24.9)	7914 (25.8)
≥25.0 kg/m2	5886 (38.4)	11656 (38.0)
**Alcohol consumption, n (%)**		
Yes	7000 (45.7)	14431 (47.1)
No	8327 (54.3)	16223 (52.9)
**Cigarette smoking, n (%)**		
Non-smoker	8580 (56.0)	18088 (59.0)
Former smoker	3226 (21.1)	6569 (21.4)
Current smoker	3521 (23.0)	5997 (19.6)

Continuous variables were represented as mean ± standard deviation (SD), and categorical variables were expressed as n (%). Unless specified otherwise, data were presented as median (interquartile range). Each instance of moderate-to-vigorous physical activity (MVPA) was categorized as lasting more than 2–30 minutes, as reported in the NHIS health screening records. MVPA was then converted into a metabolic equivalent of task (MET) score based on energy expenditure from moderate and vigorous physical activities. The MET levels for MVPA were classified into four categories: (1) physically inactive (0 MET min/week), (2) insufficiently active (1 to < 500 MET min/week), (3) active (500 to < 1000 MET min/week), and (4) highly active (≥1000 MET min/week). Depression was defined as the use of antidepressant medication or diagnosis by a specialist physician (ICD-10 F32, F33).

MVPA, moderate-to-vigorous physical activity; MET, metabolic equivalent of task; NHIS, National Health Insurance Service.

^1^Medical Aid Beneficiaries were individuals who became eligible for medical benefits for the first time between 2017 and 2018.

^2^Health Insurance Beneficiaries were individuals who did not receive medical benefits until 2018

### Depression risk disparities between medical aid and health insurance beneficiaries, regardless of physical activity changes

The gap in depression risk between medical aid beneficiaries and health insurance subscribers is detailed in Table 2, segmented by PA change groups across the two examination periods, Period Ⅰ (2013–2014) and Period Ⅱ (2015–2016). In both periods, medical aid beneficiaries consistently exhibited a higher risk of depression compared to health insurance beneficiaries, regardless of changes in PA levels. After full adjustment, in the group that was physically inactive in both Period I and Period II, medical aid beneficiaries had a significantly higher risk of depression (1.68 times) compared to health insurance beneficiaries, who were also physically inactive in both periods (aOR, 1.68; 95% CI, 1.37–2.05). In the group that was physically inactive in Period I but participated in MVPA 3–4 times per week in Period II, medical aid beneficiaries had a significantly higher risk of depression (3.33 times) compared to health insurance beneficiaries with similar physical activity changes (aOR, 3.33; 95% CI, 1.72–6.43) (Table 2). Similarly, among those who engaged in MVPA 1–2 times per week in Period I and increased to ≥5 times per week in Period II, medical aid beneficiaries had a significantly higher risk of depression (2.64 times) compared to health insurance beneficiaries with the same increase in physical activity (aOR, 2.64; 95% CI, 1.35–5.15). In the group that engaged in MVPA 3–4 times per week in both Period I and Period II, medical aid beneficiaries had a significantly higher risk of depression (2.83 times) compared to health insurance beneficiaries who maintained similar physical activity levels (aOR, 2.83; 95% CI, 1.35–5.94). Finally, in the group that engaged in MVPA 3–4 times per week in Period I and increased to ≥5 times per week in Period II, medical aid beneficiaries had a significantly higher risk of depression (2.09 times) compared to health insurance beneficiaries with the same increase in physical activity (aOR, 2.09; 95% CI, 1.10–3.98). This pattern was consistent with the findings from the Cox regression model (see S6 Table in the Supplementary Material) and the explanatory variable MET score (see S5, S6 Tables in the Supplementary Material). Table 3 categorizes patterns of PA (Physical Activity) changes between two consecutive screening periods into four groups: Continuously physically inactive, Increased physical activity, Decreased physical activity, and Continuously highly physically active. It compares the risk of depression between medical aid beneficiaries and health insurance beneficiaries, illustrating the association between receiving medical aid and the risk of depression. This study conducted two analytical models: one adjusted for gender and age (Model 1) and the other fully adjusted (Model 2). Both models consistently demonstrated that individuals receiving medical aid had a persistently higher risk of experiencing depressive episodes compared to those with health insurance, regardless of changes in physical activity. However, it was not statistically significant that medical aid beneficiaries in the continuously highly physically active group had a higher risk of depression compared to health insurance beneficiaries in the same group during both periods.

**Table 2:** Association of changes in MVPA between two biennial health screening periods (2013 to 2014 and 2015 to 2016) with risk of depression.

	Event/total	Age-sex-adjusted OR (95% CI)		Multivariable-adjusted OR (95% CI)^a^	
		Medical Aid Beneficiaries^1^	Health Insurance Beneficiaries^2^	Medical Aid Beneficiaries^1^	Health Insurance Beneficiaries^2^
**Physically inactive at the health screening Period I (2013–2014)**					
Persistently physically inactive	451/12397	1.83 (1.51–2.21)^d^	1.00 (reference)	1.68 (1.37–2.05)^d^	1.00 (reference)
Physically inactive to 1–2 times of MVPA/week	70/3193	1.39 (0.85–2.25)	1.00 (reference)	1.21 (0.73–2.01)	1.00 (reference)
Physically inactive to 3–4 times of MVPA/week	46/2091	3.90 (2.11–7.24)^d^	1.00 (reference)	3.33 (1.72–6.43)^d^	1.00 (reference)
Physically inactive to ≥5 times of MVPA/week	93/2799	1.77 (1.17–2.68)^c^	1.00 (reference)	1.57 (1.01–2.44)	1.00 (reference)
**1–2 times of MVPA at the health screening Period I (2013–2014)**					
Decrease of MVPA from 1–2 times to physically inactive	80/2870	1.41 (0.90–2.22)	1.00 (reference)	1.35 (0.84–2.18)	1.00 (reference)
Persistently 1–2 times of MVPA/week	40/2610	1.16 (0.59–2.27)	1.00 (reference)	1.10 (0.55–2.22)	1.00 (reference)
Increase of MVPA from 1–2 times to 3–4 times of MVPA/week	30/1605	1.36 (0.64–2.90)	1.00 (reference)	1.20 (0.55–2.60)	1.00 (reference)
Increase of MVPA from 1–2 times to ≥5 times of MVPA/week	40/1413	2.98 (1.57–5.64)^d^	1.00 (reference)	2.64 (1.35–5.15)^c^	1.00 (reference)
**3–4 times of MVPA at the health screening Period I (2013–2014)**					
Decrease of MVPA from 3–4 times to physically inactive	54/1838	1.57 (0.91–2.72)	1.00 (reference)	1.71 (0.95–3.08)	1.00 (reference)
Decrease of MVPA from 3–4 times to 1–2 times of MVPA/week	25/1464	0.81 (0.34–1.96)	1.00 (reference)	0.73 (0.29–1.81)	1.00 (reference)
Persistently 3–4 times of MVPA/week	33/1723	2.74 (1.36–5.53)^c^	1.00 (reference)	2.83 (1.35–5.94)^c^	1.00 (reference)
Increase of MVPA from 3–4 times to ≥5 times of MVPA/week	41/1875	2.06 (1.10–3.84)^b^	1.00 (reference)	2.09 (1.10–3.98)^b^	1.00 (reference)
**≥5 times of MVPA at the health screening Period I (2013–2014)**					
Decrease of MVPA from ≥5 times to physically inactive	101/2590	1.74 (1.16–2.61)^c^	1.00 (reference)	1.55 (1.01–2.40)	1.00 (reference)
Decrease of MVPA from ≥5 times to 1–2 times of MVPA/week	24/1084	0.85 (0.35–2.08)	1.00 (reference)	1.18 (0.46–3.05)	1.00 (reference)
Decrease of MVPA from ≥5 times to 3–4 times of MVPA/week	35/1757	1.54 (0.77–3.11)	1.00 (reference)	1.44 (0.67–3.01)	1.00 (reference)
Persistently ≥5 times of MVPA/week	102/4592	1.44 (0.95–2.18)	1.00 (reference)	1.36 (0.89–2.09)	1.00 (reference)

The adjusted odds ratio (aOR) was computed through multivariate adjusted logistic regression and reported with a 95% confidence interval (CI). Each instance of moderate-to-vigorous physical activity (MVPA) was defined as lasting more than 2–30 minutes based on self-reported NHIS health screening records. MVPA was converted into a metabolic equivalent of task (MET) score using energy expenditure from both moderate and vigorous physical activities. Categorization of MVPA levels in MET was as follows: (1) physically inactive (0 MET min/week), (2) insufficiently active (1 to < 500 MET min/week), (3) active (500 to < 1000 MET min/week), and (4) highly active (≥1000 MET min/week). Depression was defined as the use of any antidepressant medication or diagnosis by a specialist physician (ICD-10 F32, F33).

MVPA, moderate-to-vigorous physical activity; MET, metabolic equivalent of task; OR, odds ratio; CI, confidence interval; aOR, adjusted odds ratio.

^a^Adjustments were made for age, sex, household income, baseline comorbidities (hypertension, diabetes, dyslipidemia), cigarette smoking, body mass index, moderate-to-vigorous physical activity, and Charlson Comorbidity Index.

Significance levels: ^b^*P* < 0.05, ^c^*P* < 0.01, ^d^*P* < 0.001.

^1^Medical Aid Beneficiaries were individuals who became eligible for medical benefits for the first time between 2017 and 2018.

^2^Health Insurance Beneficiaries were individuals who did not receive medical benefits until 2018

**Table 3:** Association of changes in physical activity between 2013–2014 and 2015–2016 with Depression Risk among Medical Beneficiaries and Health Insurance Beneficiaries.

		Age-sex-adjusted OR (95% CI)		P value	Multivariable-adjusted OR (95% CI)^a^		P value
	Event/total	Medical Aid Beneficiaries^1^	Health Insurance Beneficiaries^2^		Medical Aid Beneficiaries^1^	Health Insurance Beneficiaries^2^	
**Continuously physically inactive from period I (2013–2014) to period II (2015–2016)**							
MET/week	451/12397	1.83 (1.51–2.21)	1.00 (ref)	<.001	1.68 (1.37–2.05)	1.00 (ref)	<.001
**Increased physical activity from period I (2013–2014) to period II (2015–2016)**							
MET/week	308/12046	1.98 (1.58–2.48)	1.00 (ref)	<.001	1.79 (1.41–2.27)	1.00 (ref)	<.001
**Decreased physical activity from period I (2013–2014) to period II (2015–2016)**							
MET/week	317/10938	1.43 (1.14–1.80)	1.00 (ref)	0.002	1.37 (1.08–1.75)	1.00 (ref)	0.010
**Continuously highly physically active from period I (2013–2014) to period II (2015–2016)**							
MET/week	13/611	3.29 (1.08–9.99)	1.00 (ref)	0.04	3.24 (0.99–10.62)	1.00 (ref)	0.06

The adjusted odds ratio (aOR) was computed through multivariate adjusted logistic regression and reported with a 95% confidence interval (CI). Each instance of moderate-to-vigorous physical activity (MVPA) was defined as lasting more than 2–30 minutes based on self-reported NHIS health screening records. MVPA was converted into a metabolic equivalent of task (MET) score using energy expenditure from both moderate and vigorous physical activities. Categorization of MVPA levels in MET was as follows: (1) physically inactive (0 MET min/week), (2) insufficiently active (1 to < 500 MET min/week), (3) active (500 to < 1000 MET min/week), and (4) highly active (≥1000 MET min/week). Depression was defined as the use of any antidepressant medication or diagnosis by a specialist physician (ICD-10 F32, F33).

^a^Adjustments were made for age, sex, household income, baseline comorbidities (hypertension, diabetes, dyslipidemia), cigarette smoking, body mass index, moderate-to-vigorous physical activity, and Charlson Comorbidity Index.

MVPA, moderate-to-vigorous physical activity; MET, metabolic equivalent of task; OR, odds ratio; CI, confidence interval; aOR, adjusted odds ratio.

^1^Medical Aid Beneficiaries were individuals who became eligible for medical benefits for the first time between 2017 and 2018.

^2^Health Insurance Beneficiaries were individuals who did not receive medical benefits until 2018.

Across the overall PA patterns, medical aid beneficiaries consistently faced higher depression risks, with risk increases of 1.80 times for increased activity, 1.68 times for continuous inactivity, and 1.34 times for decreased activity compared to health insurance beneficiaries with the same PA change patterns. However, in the consistently very active group, no significant difference in the risk of depression was observed between the two groups. Medical aid beneficiaries exhibited a higher risk of depression compared to health insurance beneficiaries even when accounting for various individual-level factors (e.g., different PA patterns, drinking habits, obesity, smoking status), regardless of changes in PA levels.

### Stratified analysis

The stratified analysis regarding the association between PA patterns and the risk of depression incidents is presented in S1 to S4 Tables. The analysis stratified age, sex, BMI, smoking status, and CCI into the following four groups: (1) Continuously physically inactive for both periods, (2) Increased MVPA from period Ⅰ to Ⅱ, (3) Decreased MVPA from period Ⅰ to Ⅱ, and (4) Continuously highly physically active for both periods (≥5 times per week). Throughout various categories such as age, sex, BMI, smoking status, and CCI, the estimated risk of depression between health insurance beneficiaries and medical aid beneficiaries generally remained consistent in subgroup analyses. However, the statistical significance tends to be attenuated in each category.

## Discussion

In this study of medical aid beneficiaries and health insurance beneficiaries, regardless of PA level changes, medical aid beneficiaries were associated with a significantly higher risk of depression. The disparity in depression risk between the two groups appeared consistent across various PA patterns, with the exception of those who maintained high levels of physical activity in both periods.

Our observation of higher depression risk among medical aid beneficiaries compared to health insurance beneficiaries, regardless of changes in PA levels, aligns with established evidence of greater mental health risk in disadvantaged social groups. A recent non-systematic review identified numerous associations linking lower socioeconomic status in specific areas to adverse mental health outcomes such as psychological distress, depression, and suicide [11]. Meanwhile, a recent meta-analysis of 49 prospective studies encompassing approximately 267,000 individuals found that higher levels of PA were associated with a decreased risk of developing depression across various age groups(OR = 0.83, 95% CI 0.79–0.88) [24]. Moreover, a systematic review and meta-analysis of 30 randomized controlled trials found that exercise-based interventions reduced depressive symptoms in people without clinical depression, with a small-to-moderate effect size [25]. However, our study adds to the existing literature by examining the differential effects of PA on depression risk across socioeconomic groups, namely medical aid beneficiaries and health insurance beneficiaries.

Previous studies have suggested that PA may moderate the relationship between lower SES and depression, but the evidence is scarce and inconsistent [26,27]Our study provides robust evidence that medical aid beneficiaries, who are more likely to experience poverty, social exclusion, and poor access to health care[16,28], have a higher depression risk than health insurance beneficiaries, regardless of their PA levels. Moreover, our study shows that only consistent high PA can reduce the depression risk among medical aid beneficiaries, while changes in PA levels do not seem to have a protective effect. This suggests that PA may not be sufficient to overcome the adverse effects of socioeconomic disadvantage on mental health, and that other interventions are needed to address the underlying causes of depression among this vulnerable group.

Socioeconomic status (SES) is a major determinant of both PA and depression, as it reflects the availability and accessibility of resources, opportunities, and social support that affect physical and mental health [29,30]. SES may affect the association between PA and depression in different ways, depending on the behavioral and biological aspects of physical activity [31]. Behaviorally, SES may influence the type, intensity, frequency, and duration of the activity, as well as the perceived benefits, barriers, and motivations for engaging in it [32,33]. Biologically, SES may influence the levels of stress, inflammation, and cortisol, [34] which are key mediators of the effects of PA on depression [35]. Lower SES is associated with higher levels of chronic inflammation, as measured by markers such as C-reactive protein (CRP), interleukin-6 (IL-6), and tumor necrosis factor-alpha (TNF-α), which may contribute to the development and maintenance of depressive symptoms [35]. Physical activity, on the other hand, has anti-inflammatory effects and can reduce the risk and severity of depression by modulating the biological pathways linking SES and inflammation [31,36]. However, the association between PA and depression may vary depending on SES, as lower SES individuals may face more barriers and challenges to engaging in regular and sufficient physical activity, as well as experiencing more negative social interactions that can trigger inflammatory responses and worsen the mood [31,37]. In fact, our study found that PA may not be sufficient to counteract the negative effects of socioeconomic disadvantage on mental health, such as poverty, social exclusion, and poor access to health care [38]. These factors may impair the antidepressant mechanisms of PA and worsen the symptoms of depression [39].

To our knowledge, this is the first study to explore whether physical activity can mediate the depression risk gap between different SES groups, represented by medical aid beneficiaries and health insurance beneficiaries, within a large population-based cohort. The observational nature of our study prevents any definitive conclusion on the causal mechanisms underlying the differential effects of PA on depression by SES. Therefore, the evidence on the effectiveness and feasibility of PA interventions for reducing depression among medical aid beneficiaries should be further supported by controlled, randomized studies.

Notable strengths of our study include the analysis of large-scale data on medical aid beneficiaries, utilizing a customized NHID that contained reliable data and objective measures of PA and depression to address the longitudinal changes in both variables. Furthermore, we successfully addressed a wide range of confounders related to sociodemographic factors, health status, health behavior, and clinical characteristics in our adjustment and stratified analyses, thereby minimizing potential bias in the study.

## Limitations

Our study has some limitations that should be acknowledged. First, we used self-report surveys to measure MVPA, which may introduce information bias or misclassification due to recall errors, social desirability, or inaccurate reporting [40]. Although we used the responses to categorize MVPA intensity and frequency and calculated MET scores to enhance the robustness of our results [41]. Second, we used the diagnosis codes and prescription records for antidepressant medications from the NHIS as a proxy for diagnosis and treatment, which may not capture the full spectrum of depression severity, duration, and treatment. Moreover, the diagnosis codes may also be prone to underreporting, especially among medical aid beneficiaries who may have limited access to mental health care [16]. Nevertheless, previous studies have validated the use of NHIS data for identifying depression cases and symptoms. Third, Additionally, the development of depression is a complex process influenced by various factors, including socioeconomic environments and health status. Certain conditions limit the evaluation of the association between physical activity and the risk of depression. For example, disabled patients who cannot engage in physical activity may have a higher risk of depression due to their underlying disability, which could contribute to an overestimation of the risk in the physically inactive group. A previous study found that hearing impairment is associated with a higher risk of depression, potentially due to reduced outdoor activity, which contributes to a higher risk of depression [42]. Due to limitations in the NHIS data, we could not adjust for disability status, which may be a major limitation of this study. Fourth, there is a challenge in distinguishing between the types of medical benefits, even though there is a possibility of differences in depression patterns between Medical Aid type 1 (targeted at individuals unable to work) and Medical Aid type 2 (created for those capable of working). However, our study is the first to investigate the differential effects of physical activity on depression by matching the entire dataset of medical aid beneficiaries representing the low SES group with health insurance beneficiaries in a large population-based cohort in Korea.

## Conclusions

While promoting physical activity remains important, our findings suggest that it may not be sufficient to mitigate the adverse mental health effects associated with socioeconomic disadvantages, such as poverty, social exclusion, and limited access to healthcare. Therefore, targeted public health interventions should not only aim to increase physical activity among socioeconomically disadvantaged groups but also address the broader socioeconomic factors contributing to mental health disparities. Further research utilizing diverse data sources is essential to elucidate the causal mechanisms underlying these effects across different socioeconomic statuses.

## Supporting Information

S1 Table Subgroup analysis of persistent physical inactivity between 2013–2014 and 2015–2016 on the risk of depression among medical beneficiaries and health insurance subscribers. (DOCX)

S2 Table Subgroup analysis of increased MVPA between 2013–2014 and 2015–2016 on risk of depression among medical beneficiaries and health insurance subscribers. (DOCX)

S3 Table Subgroup analysis of decreased MVPA between 2013–2014 and 2015–2016 on the risk of depression among medical beneficiaries and health insurance subscribers. (DOCX)

S4 Table Subgroup analysis of continuously highly physically active between 2013–2014 and 2015–2016 on the risk of depression among medical beneficiaries and health insurance subscribers. (DOCX)

S5 Table Association of changes in physical activity with the risk of depression between 2013–2014 and 2015–2016 on the risk of depression among medical beneficiaries and health insurance subscribers (categorized by MVPA and MET). (DOCX)

S6 Table Cox regression model of the association of changes in MVPA between 2 Biennial health screening periods(2013–2014 and 2015–2016) with risk of depression among medical beneficiaries and health insurance subscribers. (DOCX)
