# Supplementary material for: Socioeconomic disparities in depression risk: Limitations of the moderate effect of physical activity changes in Korea
Source: PLoS One. 2025 Feb 4;20(2):e0314930. doi: 10.1371/journal.pone.0314930 (PMC11793815; doi:10.1371/journal.pone.0314930)
Supplement: S1 Table — (DOCX) [file pone.0314930.s001.docx]

**Supplementary table 1. Subgroup analysis of persistent physical inactivity between 2013-2014 and 2015-2016 on the Risk of Depression Among Medical Beneficiaries and Health Insurance Subscribers.**

| Variables | **Multivariable-adjusted OR (95% CI)^a^** | | P value | P for interaction |
| --- | --- | --- | --- | --- |
|  | **Medical Benefit Recipients^1^** | **Health Insurance Subscribers^2^** |  |  |
| **Age** | | | | 0.02 |
| ≥ 65 years | 1.49 (1.15-1.94) | 1.00 (ref) | 0.003 |  |
| < 65 years | 2.08 (1.51-2.88) | 1.00 (ref) | <.001 |  |
| **Sex** | | | | 0.36 |
| Male | 1.86 (1.36-2.54) | 1.00 (ref) | <.001 |  |
| Female | 1.54 (1.18-2.01) | 1.00 (ref) | 0.001 |  |
| **Body mass index** | | | | 0.17 |
| <18.5 kg/m2 | 1.91 (0.60-6.10) | 1.00 (ref) | 0.28 |  |
| 18.5-23.0 kg/m2 | 1.55 (1.08-2.24) | 1.00 (ref) | 0.02 |  |
| 23.0-25.0 kg/m2 | 1.69 (1.12-2.54) | 1.00 (ref) | 0.01 |  |
| ≥25.0 kg/m2 | 1.73 (1.27-2.38) | 1.00 (ref) | <.001 |  |
| **Cigarette smoking** | | | | 0.59 |
| Non-smoker | 1.61 (1.26-2.06) | 1.00 (ref) | <.001 |  |
| Former-smoker | 2.21 (1.27-3.83) | 1.00 (ref) | 0.005 |  |
| Current smoker | 1.50 (0.93-2.43) | 1.00 (ref) | 0.10 |  |
| **Charlson comorbidity index** | | | | 0.81 |
| 0 | 1.65 (1.20-2.27) | 1.00 (ref) | 0.002 |  |
| 1 | 1.89 (1.35-2.67) | 1.00 (ref) | <.001 |  |
| ≥2 | 1.43 (0.95-2.17) | 1.00 (ref) | 0.09 |  |

The adjusted odds ratio (aOR) was computed through multivariate adjusted logistic regression and reported with a 95% confidence interval (CI). Each instance of moderate-to-vigorous physical activity (MVPA) was defined as lasting more than 2-30 minutes based on self-reported NHIS health screening records. Depression was defined as the use of any antidepressant medication or diagnosis by a specialist physician (ICD-10 F32, F33).

^a^Adjustments were made for age, sex, household income, baseline comorbidities (hypertension, diabetes, dyslipidemia), cigarette smoking, body mass index, moderate-to-vigorous physical activity, and Charlson Comorbidity Index.

Acronyms: MVPA - moderate-to-vigorous physical activity; OR - odds ratio; CI - confidence interval; aOR - adjusted odds ratio.

^1^Medical Benefit Recipients were individuals who became eligible for medical benefits for the first time between 2017 and 2018.

^2^Health Insurance Subscribers were individuals who did not receive medical benefits until 2018.
