# Supplementary material for: Socioeconomic disparities in depression risk: Limitations of the moderate effect of physical activity changes in Korea
Source: PLoS One. 2025 Feb 4;20(2):e0314930. doi: 10.1371/journal.pone.0314930 (PMC11793815; doi:10.1371/journal.pone.0314930)
Supplement: S3 Table — (DOCX) [file pone.0314930.s003.docx]

**Supplementary table 3. Subgroup analysis of decreased MVPA between 2013-2014 and 2015-2016 on the Risk of Depression Among Medical Beneficiaries and Health Insurance Subscribers.**

| Variables | **Multivariable-adjusted OR (95% CI)^a^** | | P value | P for interaction |
| --- | --- | --- | --- | --- |
|  | **Medical Benefit Recipients^1^** | **Health Insurance Subscribers^2^** |  |  |
| **Age** | | | | 0.17 |
| ≥ 65 years | 1.77 (1.26-2.48) | 1.00 (ref) | <.001 |  |
| < 65 years | 1.05 (0.74-1.49) | 1.00 (ref) | 0.77 |  |
| **Sex** | | | | 0.96 |
| Male | 1.31 (0.92-1.86) | 1.00 (ref) | 0.13 |  |
| Female | 1.39 (1.00-1.93) | 1.00 (ref) | 0.05 |  |
| **Body mass index** | | | | 0.14 |
| <18.5 kg/m2 | 1.72 (0.38-7.79) | 1.00 (ref) | 0.48 |  |
| 18.5-23.0 kg/m2 | 1.47 (1.00-2.17) | 1.00 (ref) | 0.05 |  |
| 23.0-25.0 kg/m2 | 1.59 (1.00-2.53) | 1.00 (ref) | 0.05 |  |
| ≥25.0 kg/m2 | 1.16 (0.75-1.78) | 1.00 (ref) | 0.51 |  |
| **Cigarette smoking** | | | | 0.17 |
| Non-smoker | 1.21 (0.90-1.63) | 1.00 (ref) | 0.20 |  |
| Former-smoker | 1.58 (0.86-2.92) | 1.00 (ref) | 0.14 |  |
| Current smoker | 1.77 (0.97-3.21) | 1.00 (ref) | 0.06 |  |
| **Charlson comorbidity index** | | | | 0.18 |
| 0 | 1.13 (0.77-1.66) | 1.00 (ref) | 0.52 |  |
| 1 | 1.37 (0.88-2.12) | 1.00 (ref) | 0.16 |  |
| ≥2 | 1.70 (1.09-2.65) | 1.00 (ref) | 0.02 |  |

The adjusted odds ratio (aOR) was computed through multivariate adjusted logistic regression and reported with a 95% confidence interval (CI). Each instance of moderate-to-vigorous physical activity (MVPA) was defined as lasting more than 2-30 minutes based on self-reported NHIS health screening records. Depression was defined as the use of any antidepressant medication or diagnosis by a specialist physician (ICD-10 F32, F33).

^a^Adjustments were made for age, sex, household income, baseline comorbidities (hypertension, diabetes, dyslipidemia), cigarette smoking, body mass index, moderate-to-vigorous physical activity, and Charlson Comorbidity Index.

Acronyms: MVPA - moderate-to-vigorous physical activity; OR - odds ratio; CI - confidence interval; aOR - adjusted odds ratio.

^1^Medical Benefit Recipients were individuals who became eligible for medical benefits for the first time between 2017 and 2018.

^2^Health Insurance Subscribers were individuals who did not receive medical benefits until 2018.
