# Supplementary material for: Socioeconomic disparities in depression risk: Limitations of the moderate effect of physical activity changes in Korea
Source: PLoS One. 2025 Feb 4;20(2):e0314930. doi: 10.1371/journal.pone.0314930 (PMC11793815; doi:10.1371/journal.pone.0314930)
Supplement: S4 Table — (DOCX) [file pone.0314930.s004.docx]

**Supplementary table 4. Subgroup analysis of continuously highly physically active between 2013-2014 and 2015-2016 on the Risk of Depression Among Medical Beneficiaries and Health Insurance Subscribers**

| **Variables** | **Multivariable-adjusted OR (95% CI)^a^** | | P value | P for interaction |
| --- | --- | --- | --- | --- |
|  | **Medical Benefit Recipients^1^** | **Health Insurance Subscribers^2^** |  |  |
| **Age** | | | | 0.73 |
| ≥ 65 years | 1.48 (0.81-2.69) | 1.00 (ref) | 0.20 |  |
| < 65 years | 1.39 (0.74-2.63) | 1.00 (ref) | 0.31 |  |
| **Sex** | | | | 0.90 |
| Male | 1.32 (0.74-2.34) | 1.00 (ref) | 0.34 |  |
| Female | 1.34 (0.69-2.62) | 1.00 (ref) | 0.39 |  |
| **Body mass index** | | | | 0.44 |
| <18.5 kg/m2 | NA | 1.00 (ref) | NA |  |
| 18.5-23.0 kg/m2 | 1.43 (0.63-3.23) | 1.00 (ref) | 0.39 |  |
| 23.0-25.0 kg/m2 | 1.36 (0.61-3.01) | 1.00 (ref) | 0.45 |  |
| ≥25.0 kg/m2 | 1.10 (0.54-2.23) | 1.00 (ref) | 0.79 |  |
| **Cigarette smoking** | | | | 0.04 |
| Non-smoker | 1.04 (0.56-1.92) | 1.00 (ref) | 0.90 |  |
| Former-smoker | 1.18 (0.50-2.78) | 1.00 (ref) | 0.71 |  |
| Current smoker | 3.64 (1.18-11.21) | 1.00 (ref) | 0.02 |  |
| **Charlson comorbidity index** | | | | 0.54 |
| 0 | 1.15 (0.59-2.22) | 1.00 (ref) | 0.68 |  |
| 1 | 1.58 (0.76-3.28) | 1.00 (ref) | 0.22 |  |
| ≥2 | 1.66 (0.66-4.18) | 1.00 (ref) | 0.28 |  |

The adjusted odds ratio (aOR) was computed through multivariate adjusted logistic regression and reported with a 95% confidence interval (CI). Each instance of moderate-to-vigorous physical activity (MVPA) was defined as lasting more than 2-30 minutes based on self-reported NHIS health screening records. Depression was defined as the use of any antidepressant medication or diagnosis by a specialist physician (ICD-10 F32, F33).

^a^Adjustments were made for age, sex, household income, baseline comorbidities (hypertension, diabetes, dyslipidemia), cigarette smoking, body mass index, moderate-to-vigorous physical activity, and Charlson Comorbidity Index.

Acronyms: MVPA - moderate-to-vigorous physical activity; MET - metabolic equivalent of task; OR - odds ratio; CI - confidence interval; aOR - adjusted odds ratio.

^1^Medical Benefit Recipients were individuals who became eligible for medical benefits for the first time between 2017 and 2018.

^2^Health Insurance Subscribers were individuals who did not receive medical benefits until 2018.
