# Supplementary material for: Socioeconomic disparities in depression risk: Limitations of the moderate effect of physical activity changes in Korea
Source: PLoS One. 2025 Feb 4;20(2):e0314930. doi: 10.1371/journal.pone.0314930 (PMC11793815; doi:10.1371/journal.pone.0314930)
Supplement: S5 Table — (DOCX) [file pone.0314930.s005.docx]

**Supplementary table 5. Association of Changes in Physical Activity with the Risk of Depression between 2013-2014 and 2015-2016 on the Risk of Depression Among Medical Beneficiaries and Health Insurance Subscribers (Categorized by MVPA and MET)**

|  | | **Event/total** | **Multivariable-adjusted OR (95% CI)^a^** | | P value |
| --- | --- | --- | --- | --- | --- |
|  |  |  | **Medical Benefit Recipients^1^** | **Health Insurance Subscribers^2^** |  |
| **Increased physical activity** | | | | | |
| MVPA | physically inactive (start point) | 209/8083 | 1.68 (1.25-2.25) | 1.00 (ref) | <.001 |
|  | 1-2 days/week | 70/3018 | 1.90 (1.17-3.11) | 1.00 (ref) | 0.01 |
|  | 3-4 days/week | 41/1875 | 2.09 (1.10-3.98) | 1.00 (ref) | 0.02 |
| MET | Physically inactive | 209/8083 | 1.68 (1.25-2.25) | 1.00 (ref) | <.001 |
|  | MET 0-500 | 87/3299 | 2.21 (1.42-3.44) | 1.00 (ref) | <.001 |
|  | MET 500-1000 | 12/664 | 0.59 (0.11-3.22) | 1.00 (ref) | 0.54 |
| **Decreased physical activity** | | | | | |
| MVPA | ≥5 days/week (start point) | 160/5431 | 1.44 (1.02-2.02) | 1.00 (ref) | 0.04 |
|  | 3-4 days/week | 79/3302 | 1.24 (0.76-2.00) | 1.00 (ref) | 0.39 |
|  | 1-2 days/week | 80/2950 | 1.35 (0.84-2.18) | 1.00 (ref) | 0.22 |
| MET | MET 1000이상 | 52/1806 | 1.48 (0.81-2.69) | 1.00 (ref) | 0.20 |
|  | MET 500-1000 | 119/4115 | 1.32 (0.88-1.98) | 1.00 (ref) | 0.17 |
|  | MET 0-500 | 146/5017 | 1.45 (1.02-2.07) | 1.00 (ref) | 0.04 |

The adjusted odds ratio (aOR) was computed through multivariate adjusted logistic regression and reported with a 95% confidence interval (CI). Each instance of moderate-to-vigorous physical activity (MVPA) was defined as lasting more than 2-30 minutes based on self-reported NHIS health screening records. MVPA was converted into a metabolic equivalent of task (MET) score using energy expenditure from both moderate and vigorous physical activities. Categorization of MVPA levels in MET was as follows: (1) physically inactive (0 MET min/week), (2) insufficiently active (1 to <500 MET min/week), (3) active (500 to <1000 MET min/week), and (4) highly active (≥1000 MET min/week). Depression was defined as the use of any antidepressant medication or diagnosis by a specialist physician (ICD-10 F32, F33).

^a^Adjustments were made for age, sex, household income, baseline comorbidities (hypertension, diabetes, dyslipidemia), cigarette smoking, body mass index, moderate-to-vigorous physical activity, and Charlson Comorbidity Index.

Acronyms: MVPA - moderate-to-vigorous physical activity; MET - metabolic equivalent of task; OR - odds ratio; CI - confidence interval; aOR - adjusted odds ratio.

^1^Medical Benefit Recipients were individuals who became eligible for medical benefits for the first time between 2017 and 2018.

^2^Health Insurance Subscribers were individuals who did not receive medical benefits until 2018
