# Supplementary material for: Socioeconomic disparities in depression risk: Limitations of the moderate effect of physical activity changes in Korea
Source: PLoS One. 2025 Feb 4;20(2):e0314930. doi: 10.1371/journal.pone.0314930 (PMC11793815; doi:10.1371/journal.pone.0314930)
Supplement: S6 Table — (DOCX) [file pone.0314930.s006.docx]

**Supplementary Table 6. Cox Regression Model of the Association of Changes in MVPA Between 2 Biennial Health Screening Periods(2013-2014 and 2015-2016) With Risk of Depression Among Medical Beneficiaries and Health Insurance Subscribers**

|  |  |  | **Multivariable-adjusted OR (95% CI)^a^** | | P value |
| --- | --- | --- | --- | --- | --- |
|  | **Event/total** | **Person-years** | **Medical Benefit Recipients^1^** | **Health Insurance Subscribers^2^** |  |
| **Continuously physically inactive from period I (2013-2014) to period II (2015-2016)** | | | | | |
| MVPA/week | 451/12397 | 60762 | 1.67 (1.37-2.03) | 1.00 (ref) | <.001 |
| MET/week | 451/12397 | 60762 | 1.67 (1.37-2.03) | 1.00 (ref) | <.001 |
| **Increased physical activity from period I (2013-2014) to period II (2015-2016)** | | | | | |
| MVPA/week | 320/12976 | 63948 | 1.80 (1.43-2.26) | 1.00 (ref) | <.001 |
| MET/week | 308/12046 | 59327 | 1.78 (1.41-2.25) | 1.00 (ref) | <.001 |
| **Decreased physical activity from period I (2013-2014) to period II (2015-2016)** | | | | | |
| MVPA/week | 319/11683 | 57533 | 1.34 (1.06-1.69) | 1.00 (ref) | 0.01 |
| MET/week | 317/10938 | 53816 | 1.38 (1.09-1.74) | 1.00 (ref) | 0.008 |
| **Continuously highly physically active from period I (2013-2014) to period II (2015-2016)** | | | | | |
| MVPA/week | 102/4592 | 22604 | 1.36 (0.89-2.07) | 1.00 (ref) | 0.15 |
| MET/week | 13/611 | 3005 | 3.17 (1.00-10.07) | 1.00 (ref) | 0.05 |

The adjusted odds ratio (aOR) was computed through multivariate adjusted logistic regression and reported with a 95% confidence interval (CI). Each instance of moderate-to-vigorous physical activity (MVPA) was defined as lasting more than 2-30 minutes based on self-reported NHIS health screening records. MVPA was converted into a metabolic equivalent of task (MET) score using energy expenditure from both moderate and vigorous physical activities. Categorization of MVPA levels in MET was as follows: (1) physically inactive (0 MET min/week), (2) insufficiently active (1 to <500 MET min/week), (3) active (500 to <1000 MET min/week), and (4) highly active (≥1000 MET min/week). Depression was defined as the use of any antidepressant medication or diagnosis by a specialist physician (ICD-10 F32, F33).

^a^Adjustments were made for age, sex, household income, baseline comorbidities (hypertension, diabetes, dyslipidemia), cigarette smoking, body mass index, moderate-to-vigorous physical activity, and Charlson Comorbidity Index.

Acronyms: MVPA - moderate-to-vigorous physical activity; MET - metabolic equivalent of task; OR - odds ratio; CI - confidence interval; aOR - adjusted odds ratio.

^1^Medical Benefit Recipients were individuals who became eligible for medical benefits for the first time between 2017 and 2018.

^2^Health Insurance Subscribers were individuals who did not receive medical benefits until 2018.
